# Prevalence of *Toxoplasma gondii* infection among small mammals in Tatarstan, Russian Federation

**DOI:** 10.1038/s41598-021-01582-y

**Published:** 2021-11-12

**Authors:** Nikolai D. Shamaev, Eduard A. Shuralev, Oleg V. Nikitin, Malik N. Mukminov, Yuriy N. Davidyuk, Alexander N. Belyaev, Guzel Sh. Isaeva, Vasil B. Ziatdinov, Nail I. Khammadov, Regina F. Safina, Gaysha R. Salmanova, Guzel M. Akhmedova, Kamil S. Khaertynov, Taizo Saito, Katsuya Kitoh, Yasuhiro Takashima

**Affiliations:** 1 Institute of Environmental Sciences, Kazan Federal University, 18 Kremlyovskaya St, Kazan, Tatarstan Russian Federation 420008; 2 Joint Graduate School of Veterinary Sciences, Gifu University, 1-1 Yanagido, Gifu, 501-1193 Japan; 3 Kazan State Medical Academy – Russian Medical Academy of Continuous Professional Education, 36 Butlerova St, Kazan, Tatarstan Russian Federation 420012; 4 Federal Center for Toxicological, Radiation and Biological Safety, Nauchniy Gorodok-2, Kazan, Tatarstan Russian Federation 420075; 5 Kazan State Academy of Veterinary Medicine by N.E. Bauman, 35 Sibirskiy Trakt St., Kazan, Tatarstan Russian Federation 420029; 6 Institute of Fundamental Medicine and Biology, Kazan Federal University, 18 Kremlyovskaya St, Kazan, Tatarstan Russian Federation 420008; 7 Kazan Research Institute of Epidemiology and Microbiology, Rospotrebnadzor, 67 Bolshaya Krasnaya, Kazan, Tatarstan Russian Federation 420015; 8 Department of Microbiology, Kazan State Medical University, Kazan, Tatarstan Russian Federation 420012; 9 The United Graduate School of Veterinary Sciences, Gifu University, 1-1 Yanagido, Gifu, 501-1193 Japan; 10 Department of Veterinary Parasitology, Faculty of Applied Biological Science, 1–1 Yanagido, Gifu, 501-1193 Japan

**Keywords:** Ecology, Microbiology, Zoology

## Abstract

*Toxoplasma gondii* is a zoonotic parasite with a wide host range that includes humans, domestic animals and wild animals. Small mammals serve as intermediate hosts for *T. gondii* and may contribute to the persistence of this parasite in the environment. Mass mortality in wild animals and deaths in rare endemic species make the study of this parasite of growing importance. In this study, *T. gondii* infection prevalence was evaluated in brain tissues from 474 small mammals captured at 26 trapping points in urban and rural areas of Tatarstan, Russian Federation. Nested PCR was used to detect the *T. gondii* B1 gene in the samples. Overall, 40/474 samples (8.44%) showed B1 gene positivity. *T. gondii* infection among the wild small mammals trapped in the rural area was significantly higher as a whole than that of the urban area as a whole. Multivariate logistical regression analysis also showed that the trapping area (rural or urban) significantly contributed to *T. gondii* positivity. Vegetation in the trapping points, small mammal species, sex, age or distance from the trapping points to the nearest human settlements did not significantly affect *T. gondii* positivity in the sampled small mammals.

## Introduction

*Toxoplasma gondii* is one of the most wide-spread parasites in the world. With its broad-host-range, it infects almost all mammals and birds including humans, domestic animals and wild animals. Although toxoplasmosis in humans and domestic animals is not usually symptomatic^1^, mass mortality in wild animals and deaths in rare endemic species have been reported^2–7^. Hence, attempts to understand the prevalence of *T. gondii* in wild animals are warranted. In a study conducted in Panama City, it was reported that *T. gondii* prevalence among small mammals was higher than in humans and birds from the same area^8^. Cats, the definitive hosts for *T. gondii*, shed huge numbers of environmentally-resistant oocysts in their feces, and small mammals are more frequently preyed upon by domestic cats than other intermediate hosts^9^. Therefore, it is important to understand the status of *T. gondii* prevalence among wild small mammals to understand the local situation for *T. gondii* distribution.

**Figure 1:**
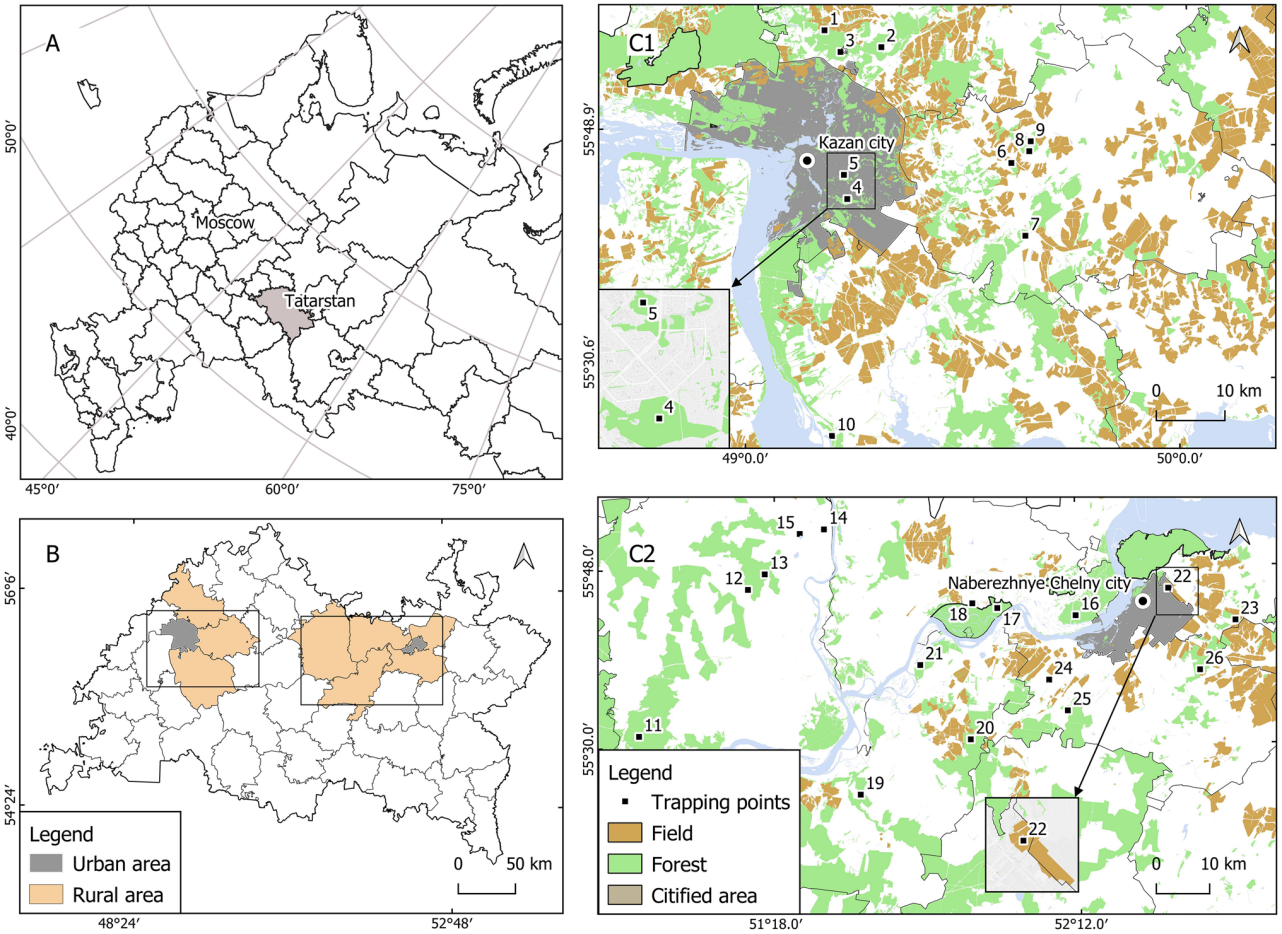
Map of the trapping points in Tatarstan, Russian Federation. (**A**) Overview map of the European part of Russian Federation showing the location of Tatarstan. (**B**) Administrative boundaries of urban (gray) and rural areas (beige). C1–C2. Spatial referencing of sampling sites: forests (green), fields (brown), citified area (gray). Locations of the urban areas appear as larger approximations in separate windows. Each trapping point is indicated by a number. Set of vector data layers was purchased from NextGIS (OpenStreetMap and contributors, 2021. Data license: ODbL. Accessed via https://data.nextgis.com).

Tatarstan, Russian Federation is located on the East European Plain in the middle reaches of the Volga River (Fig. 1A). In this area, the Volga-Kama Nature Reserve in Tatarstan contains approximately a quarter of the World’s animal species^10^. Understanding the situation regarding infectious pathogens among wild animals in Tatarstan is therefore worthwhile. We have previously reported the high seroprevalence of toxoplasmosis in humans, cats and domestic animals in Tatarstan, including in the capital city, Kazan^11,12^. However, very little is known about *T. gondii* prevalence among wild animals. Thus, in this study, we investigated *T. gondii* infection prevalence among wild small mammals in the Tatarstan.

## Results

### *T. gondii prevalence* among small mammals in Tatarstan, Russian Federation

**Figure 2:**
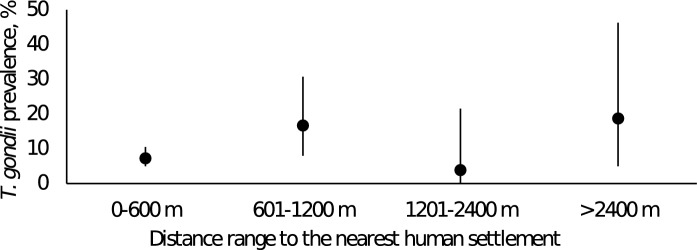
Prevalence of *T. gondii* B1-positive small mammals in each distance range to the nearest human settlement. Observed prevalence (black circles) and 95% confidence intervals (black lines) are shown.

To assess the prevalence of *T. gondii* among small mammals in Tatarstan, Russian Federation, we captured 474 of these animals at 26 trapping places (Fig. 1, Supplementary Table S1) and detected the *T. gondii* B1 gene in brain DNA from them using nested PCR. Altogether, 40/474 (8.44%, 95% confidence interval; CI: [6.16–11.4]) of the brain samples from the small mammals showed *T. gondii* B1 gene positivity (Table 1). We found that 13.56% (24/176) and 5.37% (16/298) of the small mammals trapped in the rural and urban areas showed *T. gondii* positivity, respectively, 16.66% (2/12) and 8.35% (38/455) of the individuals trapped in fields and forests, respectively, were positive, and 6.04% (9/149) and 4.16% (6/144) were male and female, respectively. *T. gondii* positivity among their ages was 8.77% (5/57) for juveniles (aged 0–2 months), 3.94% (5/127) for mature adults (aged 3–6 months), and 6.17% (10/162) for adults (aged > 6 months). Positivity among the small mammal species was 7.95% (24/302) for *Myodes glareolus* (hereafter called *My. glareolus*), 12.50% (1/8) for *Apodemus agrarius*, 11.29% (14/124) for *A. uralensis*, and 9.09% (1/11) for *A. flavicollis*. Although five *Microtus arvalis* (hereafter called *Mi. arvalis*), 13 *Sorex araneus*, and one *Dryomys nitedula* were also examined, no individuals showed *T. gondii* positivity. The distances from the trapping points to the nearest human settlements varied by 170–3890 m. As shown in Fig. 2, unlike reports from other regions^2^, no clear correlation was found between proximity to human settlements and high infection rates.

**Table 1:** *T. gondii* prevalence in small mammals trapped in Tatarstan, Russian Federation.

AreaCategory			Examined	Positive	Negative	Prevalence (%)	95% CI
**Area**	Urban	Kazan city	294	15	279	7.95	5.26–11.74
		Naberezhnye Chelny city	3	1	2	33.33	1.76–87.46
		Unknown	1	0	1	0	0–94.54
	Rural	Vysokogorsky district	22	1	21	4.54	0.24–24.88
		Pestrechinsky district	17	3	15	17.64	4.67–44.2
		Laishevsky district	16	3	13	18.75	4.97–46.3
		Mamadyshsky district	40	5	35	12.5	4.7–27.6
		Yelabuzhsky district	14	1	13	7.14	0.37–35–8
		Nizhnekamsky district	37	6	31	16.21	6.77–32.68
		Tukayevsky district	25	5	20	20	7.6–41.3
		Unknown	5	0	5	0	0–53.7
**Vegetation**	Urban	Forest	294	15	279	7.95	5.26–11.74
		Field	3	1	2	33.33	1.76–87.46
	Rural	Unknown	1	0	1	0	0–94.54
		Forest	161	23	138	14.29	9.45–20.87
		Field	9	1	8	11.11	0.58–49.33
		Unknown	6	0	6	0	0–48.32
**Sex**	Male		149	9	140	6.04	2.97–11.5
	Female		144	6	138	4.16	1.7–9.24
	Unknown		181	25	156	13.81	9.3–19.9
**Species**	*Myodes glareolus*		302	24	278	7.95	5.26–11.74
	*Apodemus uralensis*		124	14	110	11.29	6.54–18.53
	*Apodemus agrarius*		8	1	7	12.5	0.65–53.32
	*Microtus arvalis*		5	0	5	0	0–53.7
	*Sorex araneus*		13	0	13	0	0–28.34
	*Apodemus flavicollis*		11	1	10	9.09	0.47–42.88
	*Dryomys nitedula*		1	0	1	0	0–94.54
	Unknown		8	0	8	0	0–40.23
**Age**	Juveniles 0–2 months old		57	5	52	8.77	3.27–20.04
	Mature adult 3–6 months old		127	5	122	3.94	1.45–9.4
	Adult older than 6 months old		162	10	152	6.17	3.16–11.37
	Unknown		128	20	108	15.62	10–23.34
**Total**			474	40	434	8.44	6.16–11.4

### Risk factors for *T. gondii* infection

**Figure 3:**
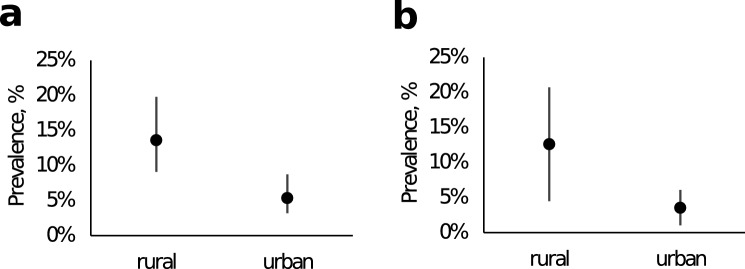
Observed and estimated *T. gondii* prevalences in rural and urban areas. (**A**) Observed prevalences among the small mammals captured in rural and urban areas. The observed prevalence and 95% CIs are shown. (**B**) Estimated prevalences from the fitted model. Values for vegetation and species were set to zero (forest and non-alien species) in the model. Estimated prevalences (black circles) and 95% CIs are shown.

We performed multivariate logistic regression analysis to separately validate the following as risk factors for *T. gondii* infection: trapping point area (urban or rural), vegetation (forest or field), small mammal species type (alien or non-alien species), age (0–2 months-old juveniles, 3–6 months-old adults or ≧ 6 months–old), sex (male or female) and distance from the trapping point to the nearest human settlement. In the classification of small mammal species, small mammal species were classified as alien (*A. agrarius, A. uralensis, A. flavicollis, Mi. arvalis*) and non-alien (*M. glareolus, S. araneus, D. nitedula*)^13^. The forward selection procedure generated a model that included area, vegetation and the type of small mammal species that best fitted the data (Model 3, Supplementary Table S2). Other factors such as age, sex and distance from trapping points to the nearest human settlements were not included in the model. In this best fitted model, the only significant factor was the area, with neither vegetation nor species deemed significant (Table 2). The model identified rural area as a risk factor (Fig. 3). The actual observed prevalence of *T. gondii* infection was also significantly higher in the rural area than the urban area (Fig. 3).

**Table 2:** Risk factors for *T. gondii* infection.

Predictor	Odds ratio (95% CI)	*p*
Area	3.92 (1.90–8.40)	< 0.001
Vegetation	–	0.138
Species	–	0.054

## Discussion

In this study, we surveyed *T. gondii* infection among small mammals in Tatarstan, Russian Federation. The overall *T. gondii* prevalence in these small mammals was 8.44%. In addition to nested-PCR targeting the B1 gene, highly sensitive *T. gondii* DNA detection methods such as the LAMP method have been reported, but the sensitivities of these methods are similar with that of nested-PCR targeting the B1 gene^14^.

It was reported that more than 30% house mice, *Mus musculus* and *Rattus rattus*, in the Omsk city, Russian Federation were infected with *T. gondii*. However, as far as we know, there is no report about *T. gondii* prevalence among wild rodents in other area of Russian Federation^15^. In several European countries (France, Czech Republic, Sweden and Switzerland) the prevalence of *T. gondii* among wild small mammals living in rural forests was 3–7%, which is not dissimilar to our results^2^. Using logistical regression analysis, we found that the trapping area (rural/urban), but not the vegetation in the trapping spots, species, sex, age or distance from the trapping points to the nearest human settlements, significantly contributed to the prevalence of *T. gondii* among the small mammals. This result is reinforced by the fact that *T. gondii* infection prevalence among the small mammals trapped in the rural area was significantly higher statistically than that in the urban area.

Although the reason why small mammals in the rural area were more frequently infected with *T. gondii* is unknown, the ranging behavior of wild small mammals in the urban and rural areas might differ, thereby affecting the possibility of their exposure to *T. gondii* oocysts. Each small mammal species has a different habitat and range^16–21^. However, in this study, similar species were captured in rural and urban areas (Supplement Fig. S1). In this study, species-specific behavior cannot explain the higher prevalence of *T. gondii* infections in the rural-captured small mammals. If small mammals in rural areas have a bigger ranging behavior than those in urban areas it could result in more frequent exposure to *T. gondii*. However, it is not yet known whether the ranging behavior of the same small mammal species in rural and urban areas differs. It will therefore be necessary to investigate the behavior of each small mammal species living in such areas.

*T. gondii* infection in small mammals might also depend on the defecation habits of stray and free-ranging domestic cats in urban and rural areas. It was reported in France that the cat feces density in rural areas is higher than that in urban areas^22^. A similar situation may also exist in Tatarstan. In the present study, *T. gondii* infection was confirmed in a small mammal captured in a rural area 3.8 km from the nearest human settlement. *T. gondii*-infected rodents were also found 2.5 km from the nearest human settlement in a rural area of London, UK^2^. In rural areas, the home-range size of free-ranging domestic cats is wider than that in urban areas^2,8–12^. Thus, it is possible that in the rural areas of the Tatarstan, Russian Federation, stray or free-ranging domestic cats deeply invade the forests and spread *T. gondii* oocysts in their fecal droppings. As far as we know, there are no reports of cat numbers or infection rates in the area and further investigation is needed.

In addition to oocyst dissemination by cats, the methods used for garbage disposal in urban and rural areas might also affect *T. gondii* infection prevalence among wild animals. Unauthorized small-scale garbage collection sites are used in the rural forests of the Tatarstan, which both free-ranging domestic cats and wild animals can freely access. Hence, cysts contained in meat-derived food waste might be an infection source in these rural areas.

In this study, we show that *T. gondii* infection among wild small mammals in the rural area is, as a whole, significantly higher than in the urban area as a whole, regardless of the former being a greater distance from human activity zones. Therefore, further research is needed to clarify the source and route of *T. gondii* transmission in wild animals.

## Methods

### Study area and sampling

Small mammals (murid rodents and shrews) were captured using mouse-type snap traps in Tatarstan, Russian Federation (Fig. 1, Table S1). Area type (urban or rural), vegetation (forest or field) and distance from trapping points to the nearest human settlement were recorded. The distinction between forest and field was made based on the UN Food and Agriculture Organization’s criteria^23,24^. Each administrative division in the Tatarstan was defined to be urban or rural by the Federal Service of State Statistics of Russian Federation^25^. Based on these criteria, Kazan city and Naberezhnye Chelny city were classified as urban districts and Vysokogorsky district, Yelabuzhsky district, Laishevsky district, Mamadyshsky district, Nizhnekamsky district, Pestrechinsky district and Tukayevsky district were classified as rural districts. Small mammals were captured during the spring and fall periods of 2016 and 2017. Fifty traps were placed in a line every 5 m in one place. Traps were baited and left for one night. Animal suffering was minimized as snap traps cause rapid death in murid rodents and shrews. Each captured small mammal’s species, age, and sex were morphologically identified using a reference guide^26^, and the animals were then stored at − 20 °C until their brains were isolated.

### Ethics

All experiments were performed in compliance with relevant Russian and Japanese and institutional laws and guidelines and were approved by the Ministry of Health of the Russian Federation and the Animal Research Committee of Gifu University (Permit Nos. MU 3.1.1029-01, and 17060, respectively). Study was carried out in compliance with the ARRIVE guidelines (https://arriveguidelines.org).

### DNA extraction and PCR

Brain tissue samples were prepared as described previously^12^. Brain samples stored at − 20 °C were transferred to a − 86 °C deep freezer. Each deep-frozen whole brain sample was homogenized in 1 ml of a 0.9% saline solution. Total DNA was extracted from the brain tissues of each small mammal using a Genomic DNA Purification Kit (Promega, Madison, WI, USA), following the manufacturer’s instructions. Nested PCR was performed with the Takara PCR Amplification Kit (Takara Bio Inc., Foster City, California, USA) according to the manufacturer's instructions. The primer sets and PCR conditions used to detect the B1 gene from *T. gondii* were those described previously^12^.

### Mapping

Spatial referencing of the sampling sites was conducted using global positioning system navigation with a Garmin eTrex 10 device. Visualization of cartographic data and measurements of the distances from the trapping points to the nearest human settlements were performed using QGIS 3.12 software^27^. Geodetic coordinates were projected into planar rectangular coordinates in the Universal Transverse Mercator projection on the WGS-84 ellipsoid (Universal Transverse Mercator, zone 39N). The overview map of the European part of Russia was made in the Lambert Conformal Conic Projection. Map coordinates are represented as geodetic coordinates (WGS-84, degrees and minutes north latitude and east longitude). To visualize thematic objects (administrative boundaries, forests, agricultural lands, and water bodies), a set of vector data layers, NextGIS (Russia), was purchased from OpenStreetMap and contributors, 2021 (https://data.nextgis.com). Data license: ODbL.

### Dataset and statistical analyses

Multivariate logistic regression was performed using the R statistical software package (version 3.6.3)^28^ to assess the trapping point area (urban or rural), vegetation (forest or field), small mammal species type (alien or non-alien species), age (0–2 months-old juveniles, 3–6 months-old adults or ≧ 6 months old), sex (male or female) and distance from trapping points to the nearest human settlements as risk factors for PCR positivity. According to previous reports^2,13,16–18^, four species, *Mi. arvalis*, *A. flavicollis*, *A. agrarius*, *A. uralensis*, and three species, *My. glareolus*, *S. araneus* and *D. nitedula* are considered alien and non-alien species, respectively. Quantitative data were replaced with 0 or 1 dummy variables, and age data were replaced by 0, 1 and 2 for juveniles, adults and elders, respectively. Multicollinearity of the explanatory variables was evaluated using Spearman’s coefficient^29^ calculated using dplyr, FSA and psych packages^30–32^. None of the Spearman’s coefficients were > 0.6. To find the best fit model, a forward selection procedure was used. Predictive performance and model fitting were assessed using the area under the receiver operating characteristic (ROC) curve, area under the curve (AUC) and corrected Akaike's information criterion (AICc) with Akaike weight (Wi). AICc and Wi were calculated using the MuMIN package^33^, and the AUC was calculated using the R pROC package^34^. *P*-values of < 0.05 were considered statistically significant. The delta method was used to compute the standard errors for the predicted probabilities based on the multinomial logit function^35^. *T. gondii* prevalence confidence intervals (95% CI) were estimated based on 468/474 samples (6 samples were excluded from analysis because they lacked information).

## Supplementary Information

Supplementary Information.

## Data Availability

The datasets generated during the current study are available from the corresponding author on reasonable request. All data analyzed during this study are included in this published article (and its Supplementary Information files).
