# Supplementary material for: Prevalence of Toxoplasma gondii infection among small mammals in Tatarstan, Russian Federation
Source: Sci Rep. 2021 Nov 12;11:22184. doi: 10.1038/s41598-021-01582-y (PMC8589860; doi:10.1038/s41598-021-01582-y)

Supplementary Information

Supplementary Table S1. Trapping site locations in Tatarstan, Russian Federation by area, vegetation type and distance to the nearest human settlement.

Trapping site	Latitude	Longitude	Description of site	Area	Vegetation	Distance (m)
1	55.953679	49.156395	Vysokogorsky district, Yash Kech village	rural	forest	170
2	55.933520	49.290105	Vysokogorsky district, Vysokaya Gora village	rural	forest	480
3	55.925886	49.195120	Vysokogorsky district, Krutushka village	rural	forest	360
4	55.733393	49.219710	Kazan city, Dubravnaya forest	urban	forest	500
5	55.764942	49.210451	Kazan city, Ometyevsky forest	urban	forest	260
6	55.785579	49.598826	Pestrechinsky district, Shikhazda village	rural	forest	1090
7	55.690598	49.634749	Pestrechinsky district, Shali village	rural	forest	580
8	55.801514	49.639878	Pestrechinsky district, Lenino Kokushkino village	rural	forest	1090
9	55.814515	49.642829	Pestrechinsky district, Lenino Kokushkino village	rural	forest	1870
10	55.422489	49.19845	Laishevsky district, Teteyevo village	rural	forest	820
11	55.521004	50.895697	Mamadyshsky district, Bersut village	rural	forest	2350
12	55.768534	51.220311	Mamadyshsky district, Verhnyaya Oshma	rural	unknown	760
13	55.794641	51.270705	Mamadyshsky district, Soty village	rural	forest	3890
14	55.869864	51.44851	Mamadyshsky district, Grishkino village	rural	forest	1820
15	55.862433	51.375958	Mamadyshsky district, Novy Kumazan village	rural	field	600

16	55.720291	52.197836	Yelabuzhsky district, Nizhnyaya Kama National Park	rural	forest	2880
17	55.734425	51.964323	Yelabuzhsky district, Tanaika village	rural	forest	200
18	55.742965	51.890115	Yelabuzhsky district, Tanaika village	rural	field	1230
19	55.422443	51.552507	Nizhnekamsky district, Sheremetyevsky Nature Reserve	rural	forest	2380
20	55.513751	51.880479	Nizhnekamsky district, Kyzylul forest	rural	forest	300
21	55.639953	51.732712	Nizhnekamsky district, Ilyinka village	rural	forest	220
22	55.746337	52.454181	Naberezhnye Chelny city	urban	field	280
23	55.707662	52.674375	Tukayevsky district, Novy village	rural	forest	470
24	55.612475	52.115869	Tukayevsky district, Biklyan village	rural	field	180
25	55.560168	52.169766	Tukayevsky district, Kalinino village	rural	forest	450
26	55.624986	52.565458	Tukayevsky district, Knyazevo village	rural	forest	1240

Supplementary Table S2. Forward selection of the fitted model

Model	Explanatory variable	AICc	Wi	AUC (95% CI)
Model 1	area	191.1	0.031	0.5551-0.7440
Model 2	area, vegetation	187.7	0.243	0.5930-0.7719
**Model 3**	**area, vegetation, species**	**186.4**	**0.458**	**0.6419-0.8062**
Model 4	area, vegetation, species, age	188.4	0.170	0.6441-0.8078
Model 5	area, vegetation, species, age, sex	190.4	0.061	0.6460-0.8091
Model 6	area, vegetation, species, age, sex, distance	192.5	0.022	0.6418-0.8061

AICc, Akaike's information criterion

AUC, area under the curve

Wi, Akaike weight

Supplementary Figure S1.

Proportional distribution of each small mammal species caught in rural (left) and urban areas (right).

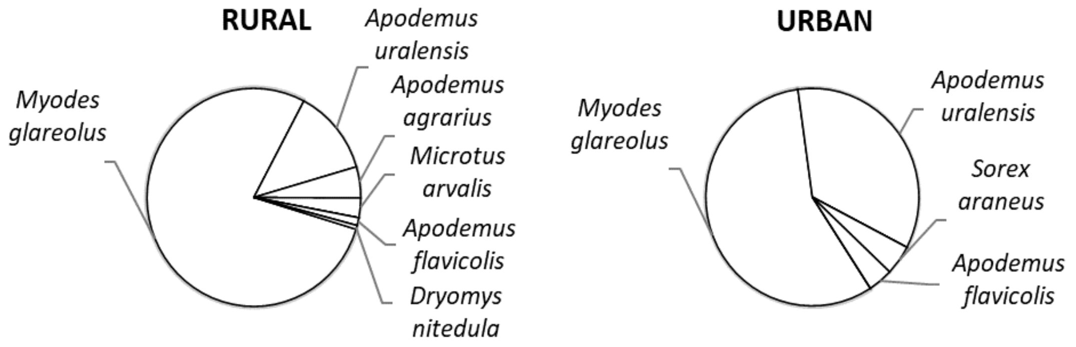

Supplement: Supplementary file 1 — Supplementary Information. [file 41598_2021_1582_MOESM1_ESM.pdf]
